# 

**Published:** 1990-09

**Authors:** 


South West Orthopoedic Club

### WEST OF ENGLAND MEDICAL JOURNAL

**SEPTEMBER 1990** **Volume 105(iii)**

Copyright © West of England Medical Journal Co Ltd

**Contents**

70 **The Ups and Downs of a Medical Referee.**
James C. Briggs.
72 **Penile Rods, The Southmead experience**
S. Gepi-Attee and J. C. Gingell.
74 **M. R. I. in Alzheimer's Disease**
J. L. G. Thomson
77 **Cervical Cancer Screening – An alternative Viewpoint**
Anthony Rogers
80 **Children of the Nineties**
Jean Golding
83 **Characteristics of HIV infection in paediatric admissions to a Rural Hospital in Zaire**
Rebecca Park
84 **Explosion in the size of the Elderly Population**
Vernon Coleman
85 **Saline Infusion and Amiloride in the management of Lithium Toxicity**
Ian Czerniewski, Jaqueline Short and A. A. McConnell
87 **Obituary** – Geoffrey Molyneux Fitzgibbon
88 **Joint Meeting of the South West Radiologists Association and the Societe d'Electro-Radiologie Medicale de l'Quest, St Malo, June 1990**
89 **Diamond Jubilee of Stoke Park Research Centre**
J. Jancar
90 **Correspondents Column**
Oman, what a challenge! Exhibition to celebrate 5 years of the Bristol University Chair of Orthopoedics. All quiet on the far Western Front. Albanian treatment for Ribbentrop's Chauffeur. Franglais a la mode moderne.
93 **Wessex Branch of the Association of Clinical Pathologists, Autumn Meeting 1989.**
95 **South West Orthopoedic Club, November Meeting 1989.**
97 **Surgical Club of the South West, November Meeting 1989.**
95 **South West Orthopoedic Club, November Meeting 1989**
97 **Surgical Club of the South West, November Meeting 1989**

*Scire est nescire, nisi id me alius sciret.* **Lucilius (180–102 BC).**

